# The effective reproductive number of the Omicron variant of SARS-CoV-2 is several times relative to Delta

**DOI:** 10.1093/jtm/taac037

**Published:** 2022-03-09

**Authors:** Ying Liu, Joacim Rocklöv

**Affiliations:** School of International Business, Xiamen University Tan Kah Kee College, Zhangzhou, 363105, China; Heidelberg Institute of Global Health & Interdisciplinary Centre for Scientific Computing, Heidelberg University, Heidelberg, 69120, Germany; Department of Public Health and Clinical Medicine, Section of Sustainable Health, Umeå University, Umeå, SE-90187, Sweden

**Keywords:** SARS-CoV-2, Omicron variant, variants of concern, B.1.1.529, Delta variant, the basic reproduction number, R_0_, effective reproduction number, R_e_

## Abstract

Our review found the effective reproduction number and basic reproduction number of the Omicron variant elicited 3.8 and 2.5 times higher transmissibility than the Delta variant, respectively. The Omicron variant has an average basic and effective reproduction number of 8.2 and 3.6.

Severe acute respiratory syndrome coronavirus 2 (SARS-CoV-2) variants of concerns (VOC) evolve in settings with high virus circulation, and are defined as variants for which there is evidence of any single or combination of following characteristics: increased transmissibility, more severe disease (e.g. increased hospitalizations or deaths), significant reduction in neutralization by antibodies generated during previous infection or vaccination, reduced effectiveness of therapeutics, vaccines or diagnostic detection failures.^1^ The newest addition to the list of VoC was Omicron [Alpha (B.1.1.7), Beta (B.1.351), Gamma (P.1), Delta (B.1.617.2) and Omicron (B.1.1.529)]. Omicron was first detected in specimens collected in Botswana and South Africa in November 2021. Although Omicron appears to be associated with less severe disease, transmissibility of this VoC is higher compared with all previous VoC. Omicron spread with an unpresented speed around the world, and by February 2022, Omicron is the dominant VoC.

We conducted a rapid review to determine the estimates of the effective and basic reproduction number (R_e_ and R_0_) of Omicron. R_e_ and R_0_ are central concepts within infectious disease epidemiology that describe the potential of epidemic spread of infectious agents. R_0/e_ is, simply put, composed of the transmissibility of a virus in combination with the contact patterns of the exposed population. R_0_ represents the average number of new infections generated by an infectious person in a totally naïve population. If R_0_ > 1, the number infected is likely to increase, and if R_0_ < 1, transmission is likely to decline and the pathogen becomes endemic and the outbreak is contained. R_e_ represents a similar quantity with the same interpretation, but in setting of non-pharmaceutical interventions and/or a background immunity in the exposed population as a result of vaccination and/or natural infection.

We accessed PubMed, Web of Science, bioRxiv and medRxiv, Google, Baidu and China National Knowledge Infrastructure (CNKI), Wanfang database and their relevant references from 1 November 2021 to 9 February 2022, using the search terms ‘B.1.1.529’ or ‘Omicron’. The inclusion criteria were studies that report the basic reproduction number or effective reproductive number of the Omicron variant, the ratio of the reproduction numbers of Omicron and Delta or give the explicit equation. Only studies published in English or Chinese were considered. The exclusion criteria were review articles, studies only investigating limited sub-populations, non-English or non-Chinese articles and studies without full text.

**Table 1:** Published estimates of R_0_ and R_e_ for the Omicron variant of SARS-CoV-2

*Relative R_0_ of Omicron to Delta*						
	First author^*^	Location	Study date	Methods	R_Omicron_ in relation to R_Delta_	
	Yu, Yangyang	South Africa	12 June 2020–1 November 2021	Estimated using susceptible-exposed-infectious-hospitalized-recovered-death (SEIHRD) model to get dynamics of Omicron, and ignored the impact of re-infection and the effects of vaccination	3.76	
	Nicolò Gozzi	South Africa	1 May–23 November 2021	Based on a compartmental model	0.25–3	
	Thomas J. Hladish	Florida, USA	December 2021	A data-driven, stochastic, discrete-time, agent based model with an explicit representation of people and places and calculating omicron’s transmission advantage as the ratio of the basic reproduction numbers of omicron and delta	2	
*Average relative basic reproduction number of Omicron in relation to Delta = 2.5*						
*Relative R_e_ of Omicron to Delta*						
	Nishiura, H.	Gauteng Province, South Africa	September–November 2021	Assume the effective reproduction number of the Omicron variant elicited 4.2 times higher transmissibility than the Delta variant in its early stage	4.2	
	Kimihito Ito	Denmark	As of 18 December 2021	The effective reproduction number of Omicron at a time point is 3.19 greater than that of Delta under the same epidemiological conditions	3.19	
	Ferenc A. Bartha	South-Africa and UK	December 2021	Employing a compartmental model of disease dynamics	4	
*Average relative effective reproduction number of Omicron in relation to Delta = 3.8*						
*Published estimates of R_0_*						
	First author	Location	Study date	Methods	R estimates	95% CI
	Huang Senzhong	South Africa	18 October–28 November 2021	Used and SEIR model and the web APP EpiSIX for the model fitting. Assumed that the mean incubation periods and infectious periods of Omicron and Delta were similar.	5.5	/
	Kaiming Bi	Worldwide	November, 2021	An artificial Intelligence model, which has been trained with tens of thousands of experimental data and extensively validated by experimental results on SARS-CoV-2	11.88	9.16–14.61
	Ferenc A. Bartha	South-Africa	December, 2021	A transmission dynamics model with pre-existing immunity, choosing population immunity in South Africa = 0 0.85	1.5–24	/
	Ferenc A. Bartha	South-Africa	December, 2021	A transmission dynamics model with pre-existing immunity, restricting the attention to a more feasible immune evasion region	1.5–13	/
	Talha Khan Burki	Worldwide	December, 2021	/	10	/
*Average R_0_ 9.5*						
*Published estimates of R_e_*						
	Kaiming Bi	25 low and middle-income countries	Prior to 5 December 2021	Immunity-based effective reproduction number, which was based on reported vaccination levels and estimates of infection-acquired immunity, and recent estimates for the transmissibility and immune-evasiveness of the Omicron variant	7.0–9.4	/
	Ontario Agency for Health Protection and Promotion	UK	As of 13 December 2021	Logistic growth model based on generation times of 5.2 days and a coefficient of variation of 2/3	3.7	3.3–4.2
	Jan-Diederik	The Netherlands	December 2021	An open-source stochastic Susceptible-Infectious-Removed fast-model	0.88	/
	Rajesh Ranjan	India	As of 10 January 2022	Using generation time versus serial interval	2.33	/
	Raquel Viana	South Africa	Early November–early December 2021	Using a phylodynamic model that accounts for variable genome sampling through time (birth-death skyline model) yields doubling times of Omicron	2.74–2.79	/
	Barnard, R.C.	England	Up to 1 December 2021	Transmission model, assuming a generation interval of 5.5 days with standard deviation 1.8 days	4	/
*Average R_e_ 3.4*						
	Dasom Kim	South Korea	November 25–December 16, 2021	Estimated using exponential growth rate and mean generation interval assumptions	1.72	1.60–1.85

^*^All the references can be found in Supplementary Materials available as Supplementary data at *JTM* online.

Search results were imported into EndNote X9 (EndNote version X9, Thomson Reuters, California), and references were screened according to the inclusion/exclusion criteria stated above. The lead author (YL) conducted the search, screened the studies by title and abstract for eligibility, reviewed the full text of the included studies and extracted the data. The articles included in this review were analysed twice by the same individual to reduce errors. The Preferred Reporting Items for Systematic reviews and Meta-Analyses guidelines were followed as illustrated in the flow diagram (Supplementary Figure S1, Supplementary data are available at *JTM* online).

In total, with the search criteria, 1914 studies were retrieved with 471 hits from PubMed, 630 hits from Web of Science, 293 through bioRxiv and 520 through medRixv. We identified 15 eligible studies of 18 estimates, which provide the basic reproductive number (8 estimates) and effective reproduction number (10 estimates) for Omicron. In Table 1, the results show that the effective reproduction number and basic reproduction number of the Omicron variant elicited 3.8 and 2.5 times higher transmissibility than the Delta variant, respectively. The Omicron variant has an average basic reproduction number of 9.5 and a range from 5.5 to 24 (median 10 and interquartile range, IQR: 7.25, 11.88). The average effective reproduction number for Omicron is 3.4 with a range from 0.88 to 9.4 (median 2.8 and IQR: 2.03, 3.85). The highest R_0_ of 24 from South-Africa is a theoretical ceiling assuming no immune evasion.

Our findings indicate that Omicron has a higher average effective, and perhaps, basic reproduction number compared with the Delta variant and the ancestral SARS-Cov-2 virus.^2^^,^^3^ The higher reproductive number of Omicron compared with Delta can be partly explained by higher intrinsic transmissibility further compounded by its immune escaping ability. The Omicron variant spread rapidly in Europe (Denmark and the UK) may be because it is capable to escape from existing population-level immunity naturally or by vaccination.

The increases in the reproductive rates of Omicron have led to a rebound of the epidemic in many countries. Preliminary estimates suggest the transmissibility may be higher among children,^4^^,^^5^ due to the fact that vaccine induced immunity is lower, and children have a much higher number and frequency of contacts. Exact estimates of R_0_ and R_e_ are difficult to determine due to confounding factors such as the extent of non-pharmaceutical interventions and prior immunity, which differs within and between countries. Variants with higher reproduction numbers such as Omicron will sweep more quickly through communities and reach the outbreak peak earlier, even if such variants will require a higher population-level immunity threshold for R_e_ to be below 1. As Omicron is antigenically the most distant to the ancestral strain, currently available coronavirus disease of 2019 vaccines have a lower vaccine effectiveness against mild infections, but still show evidence of protecting against severe disease.^6^^,^^7^

## Conclusion

The Omicron variant is spreading more rapidly than the Delta variants, likely due to a combination of increased transmissibility as measured by a higher reproduction number compared to previous variants, further compounded by its greater immune escaping ability. The reproduction number for Omicron is higher than Delta with average reproduction number of 5.08. This result is consistent with the findings of Leung GM.^8^

## Authors’ contributions

LY did the literature search and created the table and figure. JR contributed to the data analysis and interpretation. Both authors contributed to the final manuscript.

## Funding

The Youth and Middlescent Teacher Educational Research Project of Fujian Province, China (JAT200923).

## Conflict of interest

None declared.

## Supplementary Material

accepAppendix_taac037
