# Supplementary material for: The effective reproductive number of the Omicron variant of SARS-CoV-2 is several times relative to Delta
Source: J Travel Med. 2022 Mar 9;29(3):taac037. doi: 10.1093/jtm/taac037 (PMC8992231; doi:10.1093/jtm/taac037)
Supplement: accepAppendix_taac037 [file accepappendix_taac037.docx]

**Appendix**

**
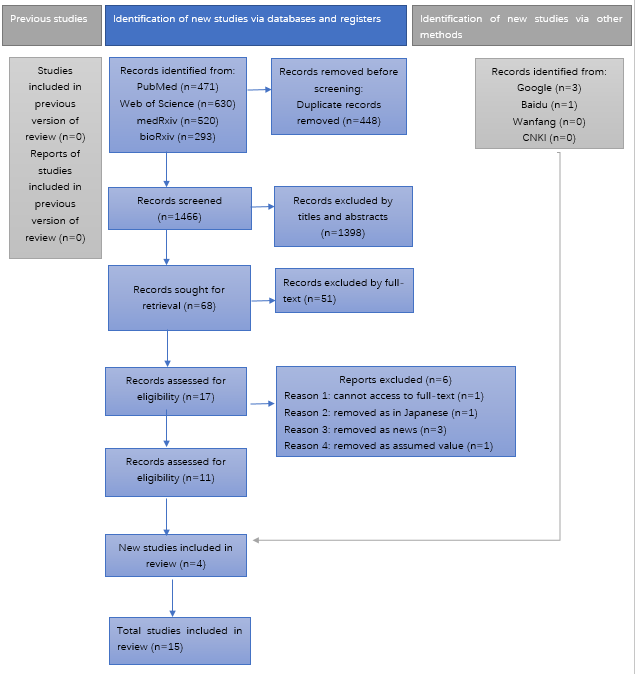
**

**Fig S1.** PRISMA 2020 flow diagram

**References**

1. Yu, Yangyang and Liu, Yuan and Zhao, Shi and He, Daihai. A Simple Model to Estimate the Transmissibility of SARS-COV-2 Beta, Delta and Omicron Variants in South Africa (December 20, 2021). Available at SSRN: <https://ssrn.com/abstract=3989919> or [http://dx.doi.org/10.2139/ssrn.3989919](https://dx.doi.org/10.2139/ssrn.3989919)
2. Nicolò Gozzi, Matteo Chinazzi, Jessica T. Davis, Kunpeng Mu, Ana Pastore y Piontti, Alessandro Vespignani, Nicola Perra. Preliminary modeling estimates of the relative transmissibility and immune escape of the Omicron SARS-CoV-2 variant of concern in South Africa. medRxiv 2022.01.04.22268721; doi: <https://doi.org/10.1101/2022.01.04.22268721>
3. Hladish TJ, Pillai AN, Longini IM. Updated projections for COVID-19 omicron wave in Florida. medRxiv [Preprint]. doi: 10.1101/2022.01.06.22268849. <https://www.ncbi.nlm.nih.gov/pmc/articles/PMC8750725/>
4. Nishiura H, Ito K, Anzai A, Kobayashi T, Piantham C, Rodríguez-Morales AJ. (2021). Relative Reproduction Number of SARS-CoV-2 Omicron (B.1.1.529) Compared with Delta Variant in South Africa. Journal Of Clinical Medicine. doi: <https://doi.org/10.3390/jcm11010030> [mdpi.com/2077-0383/11/1/30/htm](http://mdpi.com/2077-0383/11/1/30/htm))
5. Kimihito Ito, Chayada Piantham, Hiroshi Nishiura. Ito K, Piantham C, Nishiura H. Relative instantaneous reproduction number of OmicronSARS‐CoV‐2 variant with respect to the Delta variant inDenmark. J Med Virol. 2022;1‐4. doi:10.1002/jmv.275604|ITOET AL.
6. Ferenc A. Bartha, Péter Boldog, Tamás Tekeli, Zsolt Vizi, Attila Dénes, Gergely Röst. Potential severity, mitigation, and control of Omicron waves depending on pre-existing immunity and immune evasion. medRxiv 2021.12.15.21267884; doi: https://doi.org/10.1101/2021.12.15.21267884
7. Dasom Kim, Jisoo Jo, Jun-Sik Lim, Sukhyun Ryu. Serial interval and basic reproduction number of SARS-CoV-2 Omicron variant in South Korea. medRxiv: 2021.2012.2025.21268301. doi: https://doi.org/10.1101/2021.12.25.21268301
8. Huang Senzhong team of Nankai University. Identification of Omicron variant of COVID-19. <https://riph.nankai.edu.cn/2021/1129/c23093a417650/pagem.htm>
9. Kaiming Bi, Jose Luis Herrera-Diestra, Yuan Bai, Zhanwei Du, Lin Wang, Graham Gibson, Maureen Johnson-Leon, Spencer J. Fox, Lauren Ancel Meyers. The risk of SARS-CoV-2 Omicron variant emergence in low and middle-income countries (LMICs). medRxiv 2022.01.14.22268821; doi: https://doi.org/10.1101/2022.01.14.22268821.
10. Talha Khan Burki. Omicron variant and booster COVID-19 vaccines. The Lancet. doi:https://doi.org/10.1016/S2213-2600(21)00559-2. https://www.thelancet.com/journals/lanres/article/PIIS2213-2600(21)00559-2
11. Ontario Agency for Health Protection and Promotion (Public Health Ontario). COVID-19 variant of concern Omicron (B.1.1.529): risk assessment, December 13, 2021. Toronto, ON: Queen's Printer for Ontario; 2021. <https://www.publichealthontario.ca/-/media/documents/ncov/voc/2021/12/covid-19-omicron-b11529-risk-assessment-dec-13.pdf?sc_lang=en>.
12. Jan-Diederik van Wees, Martijn van der Kuip, Sander Osinga, Bart Keijser, David van Westerloo, Maurice Hanegraaf, Maarten Pluymaekers, Olwijn Leeuwenburgh, Logan Brunner, Marceline Tutu van Furth. SIR model for assessing the impact of the advent of Omicron and mitigating measures on infection pressure and hospitalization needs

medRxiv 2021.12.25.21268394; doi: https://doi.org/10.1101/2021.12.25.21268394

1. Rajesh Ranjan. Omicron Impact in India: Analysis of the Ongoing COVID-19 Third Wave Based on Global Data. medRxiv 2022.01.09.22268969; doi: <https://doi.org/10.1101/2022.01.09.22268969>.
2. Raquel Viana, Sikhulile Moyo, Daniel G Amoako, Houriiyah Tegally, Cathrine Scheepers, Christian L Althaus, Ugochukwu J Anyaneji, Phillip A Bester, Maciej F Boni, Mohammed Chand, Wonderful T Choga, Rachel Colquhoun, Michaela Davids, Koen Deforche, Deelan Doolabh, Susan Engelbrecht, Josie Everatt, Jennifer Giandhari, Marta Giovanetti, Diana Hardie, Verity Hill, Nei-Yuan Hsiao, Arash Iranzadeh, Arshad Ismail, Charity Joseph, Rageema Joseph, Legodile Koopile, Sergei L Kosakovsky Pond, Moritz UG Kraemer, Lesego Kuate-Lere, Oluwakemi Laguda-Akingba, Onalethatha Lesetedi-Mafoko, Richard J Lessells, Shahin Lockman, Alexander G Lucaci, Arisha Maharaj, Boitshoko Mahlangu, Tongai Maponga, Kamela Mahlakwane, Zinhle Makatini, Gert Marais, Dorcas Maruapula, Kereng Masupu, Mogomotsi Matshaba, Simnikiwe Mayaphi, Nokuzola Mbhele, Mpaphi B Mbulawa, Adriano Mendes, Koleka Mlisana, Anele Mnguni, Thabo Mohale, Monika Moir, Kgomotso Moruisi, Mosepele Mosepele, Gerald Motsatsi, Modisa S Motswaledi, Thongbotho Mphoyakgosi, Nokukhanya Msomi, Peter N Mwangi, Yeshnee Naidoo, Noxolo Ntuli, Martin Nyaga, Lucier Olubayo, Sureshnee Pillay, Botshelo Radibe, Yajna Ramphal, Upasana Ramphal, James E San, Lesley Scott, Roger Shapiro, Lavanya Singh, Pamela Smith-Lawrence, Wendy Stevens, Amy Strydom, Kathleen Subramoney, Naume Tebeila, Derek Tshiabuila, Joseph Tsui, Stephanie van Wyk, Steven Weaver, Constantinos K Wibmer, Eduan Wilkinson, Nicole Wolter, Alexander E Zarebski, Boitumelo Zuze, Dominique Goedhals, Wolfgang Preiser, Florette Treurnicht, Marietje Venter, Carolyn Williamson, Oliver G Pybus, Jinal Bhiman, Allison Glass, Darren P Martin, Andrew Rambaut, Simani Gaseitsiwe, Anne von Gottberg, Tulio de Oliveira. Rapid epidemic expansion of the SARS-CoV-2 Omicron variant in southern Africa. medRxiv 2021.12.19.21268028; doi: <https://doi.org/10.1101/2021.12.19.21268028>
3. Barnard, R.C., Davies, N.G., Carl A. B. Pearson, Mark Jit, W. John Edmunds. Modelling the potential consequences of the Omicron SARS-CoV-2 variant in England.  Available online: <https://cmmid.github.io/topics/covid19/omicron-england>
